# Supplementary material for: Optimization of mine ventilation network feature graph
Source: PLoS One. 2020 Nov 16;15(11):e0242011. doi: 10.1371/journal.pone.0242011 (PMC7668600; doi:10.1371/journal.pone.0242011)
Supplement: S1 File — (PDF) [file pone.0242011.s001.pdf]

Certificate of English Language Editing

Manuscript Title:

Study on optimization of mine ventilation network feature graph

Date of Revision

July 10, 2020

Abstract:

A ventilation network feature graph is the best way to directly and quantitatively reflect the features of a ventilation network. To ensure the stability of airflow in a mine and improve ventilation system analysis, we propose a new algorithm to draw ventilation network feature graphs. The independent path method serves as the algorithm's main frame, and an improved adaptive genetic algorithm is embedded so that the graph may be drawn better. Through the analysis of the topological relation of a ventilation network, a simplified mathematical model is constructed, and network simplification technology makes the drawing concise and hierarchical. A mathematical model based on the node adjacency matrix method for unidirectional circuit discrimination is constructed as the drawing algorithm may not be valid in such cases. By modifying the edge-seeking strategy, the improved depth-first search algorithm can be used to determine all of the paths in the ventilation network...

This document certifies that the manuscript listed above was copy edited for proper English language at LetPub. All of our language editors are native English speakers with long-term experience in editing scientific and technical manuscripts. We are committed to leveling the playing field for researchers whose native language is not English.

- Neither the research content nor the authors' intended meaning were altered in any way during the editing process.
- Documents receiving this certification should be considered ready for publication where language issues are concerned. *However, the authors may accept or reject LetPub's suggestions and changes at their own discretion.*
- If you have any questions or concerns about this edited document, please contact us at support@letpub.com

LetPub Your Manuscript Editor

LetPub is an author service brand owned and operated by Accdon LLC. Headquartered in the Boston area, we are a full-spectrum author services company with a large team of US-based certified language and scientific editors, ISO 17001 accredited translators, and professional scientific illustrators and animators. We advocate ethical publication practices and are an official member of the Committee on Publication Ethics (COPE).

For more information about our company, services, and partnership programs, please visit www.letpub.com.

© 2020 Accdon, LLC. All Rights Reserved. Tel: 1-781-202-9968 Email: info@accdon.com Address: 400 Fifth Ave, Suite 530, Waltham, MA 02451, United States
