# Supplementary material for: Optimization of mine ventilation network feature graph
Source: PLoS One. 2020 Nov 16;15(11):e0242011. doi: 10.1371/journal.pone.0242011 (PMC7668600; doi:10.1371/journal.pone.0242011)
Supplement: Attachment — Submitted filename: Response to Reviewer1#.docx [file pone.0242011.s002.docx]

**Response to Reviewer #1**

（1） **The last paragraph in the Introduction section needs to be revised to express the contribution of the paper better. And this contribution should be aligned with those in the abstract and other place(s).**

**Response 1:**

In order to keep the paper consistent, the author made the following modifications according to the suggestion of reviewer:

With the increase of mining depths and the expansion of mining’s scale[1], mine ventilation systems are becoming larger and their topology more complex, which makes their simulation, optimization, and prediction more difficult[2,3]. To meet the needs of modern, scientific management of ventilation systems, optimization of the drawing methods of complex ventilation networks is necessary. The ventilation network feature graph (also known as a Q-H graph) is a new way to directly and quantitatively represent the state of a ventilation system, and is an effective tool to research complex ventilation networks [4].

Euler established graph theory in the 18th century with the study of the Königsberg seven-bridge problem. In the 19th century, Guthrie made the four-color conjecture, which became the driving force in the development of graph theory[5,6]. In the 20th century, the random graph theory of Erdos and Renyi was a great theoretical leap [7]. In recent years, the research results of two-dimensional graphs have been widely applied to draw mine ventilation network graphs [8]. Mine ventilation system graphs and network graphs currently see much study, resulting in remarkable progress in algorithm optimization, topology transformation, and effect visualization. Deng et al. [9] introduced the stratification method into the ventilation network drawing, and to divide the ventilation network into different levels by using "node stratification", which reduced the difficulty of solving the minimum problem of branch intersection and improved the drawing effect; Wei et al. [10] studied the topology structure characteristics of the complex ventilation system network and the process and principle of mutual transformation, and applied the research results to the drawing of the ventilation network in Sanhejian Coal mine, which proved that the results effectively improved the drawing speed; based on the idea of graph theory, Wang et al. [11] proposed the algorithm of numbering and weight value of branch nodes, and realized the stereo visualization effect of ventilation system graph. However, these graphs can only reflect topological relationships of a ventilation system, and not their quantitative relationships. The Q-H graph can effectively solve this problem. In a Q-H graph, each branch in a ventilation network is represented by a rectangular block whose width, height, and area respectively equal the wind volume, resistance, and power consumption of the branch. The rectangular blocks are arranged in accordance with the topological relation of the network to form a wind resistance equilibrium graph [4]. The Q-H graph is equivalent to the network graph, which reflects not only the relation between nodes, circuits, and branches but the air quantity, resistance, and power consumption of each branch. It is more intuitive and quantitative than the network graph [12]. Huang..[13] [preliminarily](D:/Users/%E6%9C%89%E9%81%93%E8%AF%8D%E5%85%B8/Dict/8.9.4.0/resultui/html/index.html#/javascript:;) studied the essence and application of the Q-H graph, but did not mention its drawing algorithm. Xu et al. [12,14]proposed the “four-line” feature of the Q-H graph, that is, the function of the node, circuit, cut set, and path lines. A study was conducted on the Q-H graph drawing algorithm. Exhaustive search was used for networks, and all rectangular blocks were put together according to the search process to form the Q-H graph of a ventilation network.Based on the “four-line” feature analysis of the Q-H graph. Zhou et al. [15] put forward the resistance reduction measures of the gas extraction system, which significantly improved the gas extraction effect of the drilling field. Sun.[16] analyzed the relationship between underground air leakage and spontaneous combustion of coal by the Q-H graph, determined the control range of air leakage, and worked out an optimization scheme. Through the verification of practical engineering, the air leakage in goaf decreased from 750m^3^/min to 150m^3^/min.This shows that it is feasible to control air leakage by using the Q-H graph method. Although the above researches promote the application and development of the Q-H graph, there is a lack of in-depth research on the algorithm of the Q-H graph drawing.

The key of the existing drawing method in [14] is to turn the depth-first search method of the artificial intelligence into the exhaustive method, and Q-H graph can be formed by putting all the rectangular blocks together according to the search process. As shown in Fig 1, the search process [14] is:*e*_1_→*e*_3_→*e*_7_→(*e*_3_) →*e*_8_→*e*_9_→(*e*_8_→*e*_3_→*e*_1_) →*e*_4_→*e*_5_→(*e*_4_) →*e*_6_→(*e*_4_→*e*_1_) →*e*_2_ (The procedure in parentheses is backtracking). This paper based on the idea of independent path method(IPM), the depth-first search method is used to determine all of the independent paths. Then a Q-H graph can be drawn by establishing the coordinates of the lower-left and upper-right corners of the rectangular blocks corresponding to the branches of the independent paths. As shown in Fig 1, a total of 3 independent paths were found, namely P_1_={*e*_1_,*e*_3_,*e*_7_}, P_2_={*e*_1_,*e*_4_,*e*_5_,*e*_8_,*e*_9_}, P_3_={*e*_2_,*e*_6_,*e*_9_}.For large-scale ventilation networks, the drawing method in [14] will waste a lot of time.

(a)	(b)

**Fig 1. Transformation of network graph and Q-H graph in [14].**（a）Ventilation network graph;（b）Q-H graph.

Based on the drawing model of the independent path method, this paper uses an adaptive and improved genetic algorithm to optimize the sorting problem of independent paths in order to solve the problem of the large number of divided rectangular blocks in the drawing process, so that the number of rectangular blocks divided in the QH graph is reduced. and the drawing effect is more intuitive. A mathematical model based on the node adjacency matrix method for unidirectional circuit discrimination is constructed so that the unidirectional circuit in ventilation network can be accurately identified. By modifying the edge-seeking strategy, the improved depth-first search algorithm can be used to determine all of the paths in the ventilation network with unidirectional circuits, and the equivalent transformation method of network topology relations is used to draw the ventilation network with unidirectional circuits feature graph. In addition, the application of network equivalent automatic simplification technology in Q-H graph drawing can shorten the drawing time and make the Q-H graph hierarchical. The powerful function of Q-H graph can provide a theoretical basis and technical support to intelligent diagnosis, reliability analysis, and optimization of complex ventilation network systems. It can be applied to ventilation systems such as of subways, tunnels, and large shopping malls.

（2） **There are long lists of citing works in the Introduction section without any discussion on them (e.g. [1-5], [7-9], [11-16] etc. I think it is much more useful if the authors provide at least a brief discussion or presentation on these cited works.**

**Response 2:**

According to the suggestion of reviewer, the author has revised the citation methods of the references in the paper and made a brief description of references (it has been revised and marked in the paper).

（3） **The authors have the whole long section 2 to explain about the drawing model and optimisation research of the Q-H Graph. This section also includes quite an extensive technical presentation. I feel that this is quite excessive that might confuse the reader(s) on the contribution of the paper and the existing work. Perhaps, it is a good idea to short this section and reference to other paper(s) for further information.**

**Response 3:**

According to the suggestion of reviewer, the author has made relevant modifications and abridgments to this chapter by referring to the writing methods of relevant literature, which reflects the author's main contributions to the research subject and makes the article more accessible.（The specific modification content has been marked out in the paper.）

（4） **Section 5 and section Section 6 need to be re-written. It is not a good idea to use just list points for the sections in a scientific paper.**

**Response 4:**

The author made the following modifications according to the suggestion of reviewer:

**5. Application**

The application of Q-H graph is of great value in the daily management and reconstruction of mine ventilation system. Q-H graph can be used to adjust the air quantity as needed, determine the total resistance, analyze the three-area management of mine ventilation system, map the mine air environmental parameters, map the mine pollution range and determine the escape route, etc.

**5.1 Regulation of air volume and determination of total resistance**

When the resistance and air quantity of the paths in the ventilation network are known, the amount, location, the number of paths and means of adjustment can be determined by the use of the Q-H graph. The path method is adopted to adjust (*n*-*m*+1) small resistance paths with the maximum resistance path as the benchmark, which makes the resistance of each path equal, so as to ensure the minimum power consumption. In addition, for any ventilation network, the Q-H graph can be used to determine the total resistance directly from the upper-right coordinate, without complicated calculation.

**5.2 Management of three areas**

The ventilation system can be divided into three areas: downcast air area, returning air area and using air area. The proportion of resistance of each area is an important indicator to measure the quality of the ventilation system. The proportion of resistance in three areas of natural air distribution can reflect whether the design of ventilation system is reasonable. The proportion of resistance in three areas of air distribution according to demand can reflect whether the production layout and technical management is reasonable. The Q-H graph shows the range of the three areas, as shown in Fig 20(a)..

**5.3 Display of mine gas concentration, temperature, humidity and other parameters**

The Q-H graph can intuitively display the gas concentration, temperature and humidity of each path in the mine, which is convenient for the management of the entire mine ventilation system. Fig 20(b) is the schematic diagram of gas concentration. The gas concentration is represented by different colors in the Q-H graph, and the distribution of gas concentration in the whole mine can be clearly seen.

**5.4 Display of mine pollution scope and escape route**

The Q-H graph can show the contaminated and non-contaminated areas, as shown in the Fig 20(c). When there is a mine disaster, the Q-H graph can determine the route to escape. The workers who are blocked in the mine can avoid the disaster according to the safe route shown in the graph.

(a)	(b)	(c)

**Fig 20.Functions of Q-H graph.** (a) Q-H graph of distribution of three areas; (b)Q-H graph of distribution of gas concentration; (c) Q-H graph of distribution of pollution scope.

In addition to the above functions, the Q-H graph can also be applied in the following aspects: providing parameters for pressure equating fire prevention technology[12], determining boundary conditions of seepage field[12], ventilation system reconstruction[27], showing mine ventilation facilities[27], determining extreme value flow of ventilation system[28], etc. In conclusion, the Q-H graph plays a very important role in the management of mine ventilation system.

**6. Conclusion**

In this paper, independent path method, improved adaptive genetic algorithm, the equivalent transformation of the topological relation and network simplification method are used to study the modeling, optimization, specialized processing(the ventilation network with unidirectional circuits) and level-division of Q-H graph, and the following main conclusions are drawn:

An independent path method was used to construct the mathematical model for drawing Q-H graphs. The improved adaptive genetic algorithm was used to optimize the cutting of the rectangular block in the process of Q-H graph drawing, whose optimal effect was realized, which is a good reference for the theoretical research and programming of the Q-H graph.

Based on node adjacency matrix theory, a mathematical model to identify unidirectional circuits was constructed. By modifying the edge-seeking strategy, the depth-first algorithm is suitable for both ordinary ventilation networks and those with unidirectional circuits. By using the equivalent transformation method of the network topological relation, the Q-H graph drawing of a network with unidirectional circuits was solved, and the drawing design theory of a complex mine ventilation network was enriched and improved.

The equivalent simplified mathematical model of the network was established, and the simplified process and principle were studied. Using network simplification technology can effectively improve the analysis speed and shorten the drawing time of a complex mine ventilation network, and realize the hierarchy of the Q-H graph, so it can intuitively reflect the overall features of the network and describe a local situation in detail.

The Q-H graph has a powerful practical function and has changed the traditional ventilation management methods. It is a new reform, which opens a new way for the modernization of ventilation management technology.

（5） **Minor points:**

**1）+ Figure 8: it is a good idea to link the Steps in the text after Figure 8 with the Figure. It is hard to know which steps are referring to which items in the flow charts.**

**Response 5:**

As suggested, the following modifications have been made in the paper:

**2）+ Figure 10: I think it is a bad idea to use the 3D bar graphs. The inconsistency in bar height in 2D display can easily mislead the interpretation of the values.**

**Response 6:**

+ Figure 10 has been deleted in the paper.

**3）+ Figure 12, page 12, at the caption, please fix the format problem for the axes labels**

**Response 7:**

The above problems have been revised in the paper.
